# Commentary Regarding the Effect of Long Term Use of Finasteride on Saudi Women

**DOI:** 10.31557/APJCP.2020.21.8.2197

**Published:** 2020-08

**Authors:** Wedad Saeed Al-Qahtani, Gadah Albasher, Saad Alkahtani

**Affiliations:** 1 *Department of Forensic Sciences, College of Criminal Justice, Naif Arab University of Security Sciences, Riyadh, Saudi Arabia. *; 2 *Department of Zoology, College of Science, King Saud University, Riyadh, Saudi Arabia. *

**Keywords:** Finasteride, steroids, menstrual bleeding, women

## Abstract

In the following commentary, we provided discussions regarding an article bublished by Albasher et al. (2020) concerning the effects of long-term use of finasteride on women. They reported some adverse effects, including abnormal levels in the steroid hormones, irregular menstrual cycles, and heavy menstrual bleeding. Some concerns exist in the study mainly concerning the sample, particularly its size and composition. The authors recruited Saudi women. Thus, the findings cannot be generalized. Their study can be seen as a starting point in this regard. Even though the concerns can restrict the study, their study is still credible addressing critical issues that need to be taken seriously by other researchers and stakeholders.

*Finasteride in Women*

Finasteride is a competitive 5-alpha-reductase inhibitor, an enzyme that catalyzes testosterone conversion to dihydrotestosterone (DHT). Although human body has several isotopes of 5-alpha-reductase, finasteride specifically acts on isoenzyme II present in the uterus, endometrium, fallopian tube, prostate, male genitalia, hair follicle root sheath, and liver (Drake et al., 1999). Since finasteride does not inhibit testosterone synthesis directly or interfere with the androgen receptor, its physiological activity is restricted to DHT-dependent tissues. This drug is used to treat individuals with pattern hair loss, prostate cancer, and prostatic hyperplasia because of its inhibiting effect (Oliveira et al., 2013). However, there is a few research regarding its effect on women. Albasher et al., (2020) took the initiative to study the long-term use of finasteride and its effect on women. Hair loss in female or androgenetic alopecia is a common concern in women and is challenging to treat and understand. It entails the reduction in vertex hair density, while the front hairline is not affected (Price, 2003; Messenger and Sinclair, 2006). It is understood that the condition occurs to an estimated 10 percent of pre-menopausal women, and there is a rise of 50–75 percent to menopausal women who are 65 years or older. The primary purpose of the medication is the reduction of the hair loss through limiting the conversion of testosterone to DHT by 5-αreductase type II, in a process that reduces fat tissue that is around the hair follicle, resulting in dehydration of the skin, reduction of nutrients and blood flow, and shrinkage of the hair follicle. The primary purpose that their study aimed at was discovering the effect of prolonged use of this medication on women. These adverse effects include abnormal levels in the steroid hormones, irregular menstrual cycles, and heavy menstrual bleeding (Bao et al., 2014).

There are several issues of concern to the study performed by Albasher et al., (2020) . One concern is its small sample size. Thirty Saudi women were involved in Albasher et al.’s study. Such a small number of sample is not enough to represent Saudi population. Future studies with larger sample size are needed to investigate the effect of prolonged use of finasteride-induced gonadal sex steroids alterations on menstrual bleeding in women to achieve more accurate and reliable findings. 

Another concern is the age of the women involved in the study. It is evident from their article introduction section that androgenetic alopecia occurs in 10 percent of the pre-menopausal women, which is a significant number but not as concerning as the older women that seem to be at higher risk of getting the condition. From the introduction section, it can be induced that the population that is more likely to use finasteride is the older population of women, and the researchers did not consider this issue in their study. The authors explicitly recruited women in the age range of 25 to 35, though the medication are indicated for menopausal women that are 65 years or older. From the study, it can be agreed that the findings represent the age group that was used in the study and not those that are 65 or older. The authors could involve a broader age group to provide more comprehensive findings. 

It is clear that the study by Albasher et al., (2020) is a starting point of the concerns of the medication on the female population. There is a need to conduct the study on a more diverse group. The inclusion of women with diverse age groups and cultural backgrounds can increase the variables of the study . As a result, the authors of that research could get more robust results since the menstrual cycle is one of the areas that the medication has adverse effects. 

In addition, it is important to understand that environmental factors play an essential role in determining the menstrual flow and regularity (Jahanfar, 2012). These environmental factors are the guideline regarding the suitable medication . 

In spite of limitations relating to sample size and participants’ age e, the authors did an excellent job in their study. They applied correct procedures while selecting the samples. They did appropriate data analysis and discussed the findings very well. 

Although Saudis are conservative, the researches were successful in managing this issue. Additionally, the results of the study are valid and should be taken seriously. The use of other peer-reviewed literature can increase the credibility of the study. The researchers are clear in their findings and warned women under androgenetic alopecia treatment not to use finasteride. Albasher et al., (2020) yielded that the prolonged use of medication was associated with adverse effects that are common in women under androgenetic alopecia treatment. These adverse effects are DNA damage, heavy menstrual bleeding, high cholesterol, and aromatase disorder.

Overall, the study provides compelling evidence regarding the effect of prolonged use of finasteride that should be taken seriously by the stakeholders. These stakeholders include pharmaceutical organizations, healthcare practitioners, and the women that are using the medication . Female reproductive health is an essential area in healthcare, and the study can be of great assistance in the area. The authors of the study highlights some limitations that are critical for future studies on this topic. Increasing the sample size is one of the concerns that can be removed in future studies. Furthermore, the age group should be also widen to capture different women in society. As mentioned above different environmental factors are important in such studies and should be taken in consideration for better understating the outcomes of future studies.

The dosage of 1 mg/day for prolonged duration of the drug should be considered for the study . As previously reported, 1mg of finasteride has plasma drug concentration of 9.2 ng/ml after 1-2 hours administration. The plasma half-life of finasteride is 6-8 hours and tissue binding is 4-5 days. Therefore, the pharmacokinetic study using the dosage of 5 mg/day is suggested. The following tests are recommended to be performed: blood tests cell blood count, aspartate aminotransferase, alanine transaminase (ALT), total bilirubin alkaline phosphatase, blood glucose, urea, creatinine, iron, dehydroepiandrosterone sulfate (DHEA-S), delta-4-androstenedione, 5α-DHT, 17β-prolonged, cortisol, prolactin luteinizing hormone (LH), and follicle-stimulating hormone (Albasher et al., 2020).
